# Oxytocin and oxygen: the evolution of a solution to the ‘stress of life’

**DOI:** 10.1098/rstb.2021.0054

**Published:** 2022-08-29

**Authors:** C. Sue Carter, Marcy A. Kingsbury

**Affiliations:** ^1^ Kinsey Institute, Indiana University, Bloomington, IN 47405, USA; ^2^ Department of Psychology, University of Virginia, Charlottesville, VA 22904, USA; ^3^ Lurie Center for Autism, Mass General Hospital for Children, Harvard University Medical School, Charlestown, Boston, MA 02129, USA

**Keywords:** oxygen, oxytocin, vasopressin, social behaviour, mitochondria, oxidative stress

## Abstract

Oxytocin (OT) and the OT receptor occupy essential roles in our current understanding of mammalian evolution, survival, sociality and reproduction. This narrative review examines the hypothesis that many functions attributed to OT can be traced back to conditions on early Earth, including challenges associated with managing life in the presence of oxygen and other basic elements, including sulfur. OT regulates oxidative stress and inflammation especially through effects on the mitochondria. A related nonapeptide, vasopressin, as well as molecules in the hypothalamic–pituitary–adrenal axis, including the corticotropin-releasing hormone family of molecules, have a broad set of functions that interact with OT. Interactions among these molecules have roles in the causes and consequence of social behaviour and the management of threat, fear and stress. Here, we discuss emerging evidence suggesting that unique properties of the OT system allowed vertebrates, and especially mammals, to manage over-reactivity to the ‘side effects’ of oxygen, including inflammation, oxidation and free radicals, while also supporting high levels of sociality and a perception of safety.

This article is part of the theme issue ‘Interplays between oxytocin and other neuromodulators in shaping complex social behaviours’.

## 1. Overview

‘… life is a special, very complex form of motion of matter, but this form did not always exist, and it is not separated from inorganic nature by an impassable abyss; rather, it arose from inorganic nature as a new property in the process of evolution of the world.’ [1, p.1]

Oxygen is essential to most life on Earth. However, oxygen's benefits come with a unique set of challenges which collectively helped to create ‘the stress of life’ [2]. Over time, the evolution of complex organisms brought biological and behavioural challenges that were also managed in part by a nonapeptide known as oxytocin (abbreviated here as OT) [3]. Although in an evolutionary sense OT and the OT receptor (OTR) are comparatively recent, the OT system is a component of an ancient hierarchy (table 1). The relationship between oxygen and peptide molecules, including precursors to OT [4], spans hundreds of millions of years or more.

**Table 1.** Evolutionary origins of molecules managing inflammation, stress and coping.

time	evolution of chemicals:	
Present		
>200 Ma	mammals	oxytocin
>500 Ma	vertebrates	vasopressin
<542–485 Ma	many new species	vasotocin- and CRH-like
approx. 541 Ma	the Cambrian explosion begins, with oxygen levels capable of supporting terrestrial life	
>4 Ba–541 Ma	metazoan	rudimentary hypothalamic–pituitary–adrenal axis
Pre-Cambrian	unicellular	basic biological molecules (including neuropeptides, steroids, cytokines, GPCRs)
>4.5 Ba	formation of earth	basic elements (including H, C, N, O, S)
>14 Ba (Big Bang)	formation of universe	particles and matter

OT works in conjunction with the related, but more ancient nonapeptide, vasopressin (VP) and traditional ‘stress hormones,’ including corticotropin-releasing hormone (CRH). However, as described here, many of the actions of OT are best appreciated in the context of its interactions with oxygen. The specific purpose of this narrative review is to describe those interactions [4] and their relationship to the management of challenges that have been called ‘stress.’

## 2. Origins

Life depends on primal inorganic elements. These elements are grounded in the physical properties of particles and matter. Oxygen, hydrogen, nitrogen, carbon and sulfur became building blocks for a multitude of organic molecules. The relationships among these chemicals allowed the storage of energy from the sun and the evolution, survival and reproduction of life forms that currently inhabit Earth. In this context, the evolution of life on Earth took billions of years (table 1). During that period biological systems gradually emerged capable of the management of gases (including oxygen), fluids (including water), nutrients (carbohydrates, fats, amino acids and minerals) and various mechanisms for the defence of tissues and organisms (including cytokines and immune systems). The origins and mechanisms for the diverse interactions between oxygen and the precursors for OT go back to the point at which peptides first became involved in the origins of life, estimated at approximately 3.8 billion years ago (Ba) [1]. Peptide molecules including OT-like molecules were early components of cellular defences and homeostatic mechanisms, with subsequent consequences for vertebrate development and behaviour, and the management of challenges and stressors [1,5].

## 3. Oxygen: ancient earth, the great oxidation event and the Cambrian explosion

Over a period of more than 4.5 billion years, an array of molecules merged to form planet Earth (table 1). The events that led to life on Earth are lost in time and hotly debated. However, it is generally accepted that in the presence of sunlight, two basic elements—oxygen and hydrogen—united, forming the water which eventually covered early Earth.

Most of the oxygen on Earth is believed to have been formed in the ocean by photosynthesis in cyanobacteria (blue-green bacteria). The water gradually receded and atmospheric oxygen reached a critical level, creating what has been called the ‘great oxidation event’ (GOE), dated to around 2.4 Ba [6]. The GOE is believed to have eventually supplied the oxygen necessary for the evolution of new life forms [7]. Following the GOE, the availability of atmospheric oxygen allowed the expansion of land masses, new habitats and the evolution of additional species.

About 541 million years ago (Ma), associated with the emergence of high levels of gaseous oxygen, the fossil record documents a dramatic increase in the complexity of life forms [8]. The period that followed saw the ‘Cambrian explosion,’ defined by the rapid emergence of multi-cellular organisms, including eventually millions of distinct species. Genomic evidence suggests that the precursors for modern peptides, enzymes, neurotransmitters, neuromodulators, hormones and nucleotides existed prior to the Cambrian explosion. Among these were familiar molecules, still used by contemporary animals, including glycine, glutamate, gamma amino butyric acid (GABA), acetylcholine, catecholamines (dopamine, norepinephrine and epinephrine), indolamines (serotonin and melatonin), opioids, endocannabinoids, steroids (glucocorticoids, progestins, androgens and oestrogens) and their various receptors. Although its precursors existed before the Cambrian explosion [9–13], among the post-Cambrian products of this evolutionary process was the later evolution of the comparatively ‘modern’ peptide known as OT [4,14].

Oxygen, Earth's most abundant element, is active in the physiological processes of aerobic organisms and provides the energy that supports cellular metabolism. Oxygen is a biochemically reactive molecule, capable of forming bonds with other elements. Oxygen occurs in abundance in the atmosphere as a gas, with two atoms of oxygen binding together. As a gas, oxygen forms over 20% of the Earth's atmosphere. Almost half of the Earth's crust also consists of high levels of oxygen, often in the form of oxides—molecules that contain at least one oxygen molecule, such as iron oxide. Oxygen also partners with carbon and hydrogen, storing available energy primarily in the bonds of carbohydrates and fats. During aerobic cellular respiration, energy can be released as a result of the breaking of these chemical bonds. At the end stage of aerobic cellular respiration, known as oxidative phosphorylation, oxygen is essential for electron transfer within the mitochondrial membrane for the generation of energy in the form of adenosine triphosphate (ATP).

In contemporary organisms, the OT system influences physiology and behaviour across levels of biological organization from mitochondria [15] and the microbiome [16] to motherhood [17] and monogamy [18]. For example, OT systems are implicated in unique features of mammalian reproduction, including lactation, birth and parental nurture [3]. OT and its primary receptor (OTR) had a specific role in the evolution of mammals [14,19], eventually supporting the development of unique systems necessary for human language and consciousness [20,21].

In an atmosphere dominated by oxygen, the necessity to manage this energetic molecule changed everything [22]. Oxygen is a highly reactive non-metal element that also supports oxidation and inflammation and is able to form oxides with most elements and various compounds, including the generation of free radicals. The chemical by-products of metabolism and inflammation are adaptive for cell survival as they contribute to cellular homeostatic mechanisms, yet these by-products also hold the potential to be dangerous, contributing to disease and ageing [23].

## 4. Sulfur

Prior to the abundance of oxygen, sulfur was the most important source of cellular energy for living organisms. Sulfur is believed to have originally formed in volcanos, possibly by the fusion of silicon and helium. Sulfur is also found in heat vents in the deep ocean, which have been proposed as sites for the evolution of life [7]. Sulfur continues to maintain life in anerobic organisms including some bacteria and archaea and plays a role in mitochondrial function. Interactions between oxygen and sulfur were critical to the chemistry of early organisms [24].

Sulfur-based systems exist in anerobic bacteria in the digestive system and are probably important for the protective functions of the microbiota [16]. However, sulfur-based metabolism and cellular respiration are less efficient than oxygen-based processes. Sulfur also does not diffuse as easily as oxygen through cell walls. For these and other reasons, sulfur may not be as well-suited as oxygen to the needs of multi-cellular organisms.

Oxygen and sulfur share some chemical properties. Sulfur sits in a parallel position to oxygen in the periodic table. However, sulfur has the potential for forming complex chemical configurations that do not appear in oxygen.

At appropriate levels, sulfur-containing molecules, including those in food or in the environment, continue to play anti-inflammatory and anti-oxidant roles in human health [25–27]. However, sulfur, historically known as ‘brimstone,’ is particularly volatile and potentially corrosive. A world based on sulfur, rather than oxygen, might have looked more like Dante's hell, than modern Earth.

## 5. Oxygen, sulfur and oxytocin

OT-like peptides and their receptors are believed to have evolved over 700 Ma [14,28]. The OT system and the associated benefits of sociality played a unique role in the integration of the benefits and dangers associated with oxygen. OT-like molecules filled a niche that permitted the eventual evolution of sophisticated neural systems, including the ‘brain’ and autonomic nervous systems, which became especially important to the patterns of selective sociality found in mammals [29,30].

OT interacted with, and continues to interact with, the various molecules listed above. Dynamic disulfide bonds are necessary for the biological activity of OT [31,32] and may be critical to the anti-inflammatory effects of OT or fragments of that molecule [15,33]. Chemical interactions between sulfur and oxygen also are relevant to the role of OT in the evolution of mammalian sociality [20].

OT has a central role in modulating ‘oxidative stress’—a process defined as an ‘imbalance between the generation of oxidants and local anti-oxidative defense’ [34, p. 1]. Biochemically active sulfur bonds (thiols), found in the amino acid, cysteine, are part of the biochemistry of OT-like hormones and their precursors (table 2) [4,32]. The disulfide bonds in OT may protect against excessive oxidation, inflammation [15,37,38] and pain [39,40]. During oxidative stress, disulfide bonds form what has been called a ‘bridge over troubled waters' [41, p. 1]. For example, mitochondrial thiols form a reversible network that is important for reacting to and restoring redox homeostasis occurring during oxidative stress [34].

**Table 2.** Amino acid sequences for major peptides in the OT–VP family.^a.^

amino acid position	1^b^ 2 3 4 5 6^b^ 7 8 9	expressed in (among others)^a^
vasotocin	Cys-Tyr-Ile- Gln-Asn-Cys-Pro-Arg-Gly (NH2)	non-mammalian vertebrates and fetuses
vasopressin	Cys-Tyr-Phe-Gln-Asn-Cys-Pro-Arg-Gly (NH2)	mammals
oxytocin	Cys-Tyr-Ile- Gln-Asn-Cys-Pro- Leu-Gly (NH2)	mammals
mesotocin	Cys-Tyr-Ile- Gln-Asn-Cys-Pro- Ile- Gly (NH2)	non-eutherian tetrapods and birds

^a^[35,36].

^b^Disulfide bonds between cysteines at positions 1 and 6 can form a ring in these molecules.

OT regulates reactions to both physical and social threats and especially chronic stressors and disease [42]. In mammals, OT specifically facilitates parental investment and the formation of protective and selective attachments [43,44]. OT is of particular importance during birth, nursing and perinatal life and has the capacity to epigenetically and adaptively programme early development [17,45,46]. OT has protective consequences during these events and time periods, including consequences for modulating homeostasis with effects that range from molecular to behavioural levels.

The potentially detrimental effects of oxygen also are managed in part by the anti-inflammatory and anti-oxidant properties of OT. Across many levels of function, the presence of dynamic disulfide bonds may be particularly critical during challenge or stress (reviewed in [3,4,15,47]). However, these same disulfide bonds in OT also have made the OT-like molecules difficult to study and measure especially in bodily fluids [32]. Despite these problems it is clear that interactions between compounds containing sulfur and oxygen play a critical role in the functions of peptides including OT.

## 6. The evolved power of peptides

Peptide molecules are integrated into all aspects of life. Peptides allowed living organisms to use elements including oxygen, carbon, nitrogen, hydrogen, sulfur and various metals to create amino acids [1]. They also supported growth, adaptation, protection and reproduction**.** There is evidence that among the unique powers of OT-like peptides are its capacity to regulate the inflammatory effects of oxygen [3,17]. OT also has a variety of other functions including guiding the development and functions of the brain and body and, within cells, and as described here, regulating cellular metabolism through effects on the mitochondria [15].

OT-like molecules in particular had properties that provided functions across both evolutionary time and the lifespan [3,23]. Through effects on critical functions in the nervous, autonomic and immune systems, as well as within mitochondria, OT-like molecules have had pivotal roles in homeostasis, physiology and behaviour. OT-like molecules are components of intricate and highly evolved networks which facilitate interactions with the physical and social environment. All of these functions were essential for the evolution of complex social systems [48], allowing social experiences to regulate health and wellbeing [4,29,43].

Critical points of intersection between oxygen and OT are found in the capacity to efficiently extract gases, minerals and nutrients from the environment. OT functions in part through its capacity to serve as a component of mammalian defence systems with both anti-inflammatory and anti-oxidant effects [38]. Particularly under conditions of stress, including an abnormal oxygen supply, OT may dampen the production of excessive oxidants and reactive oxygen species (ROS). This link is found in responses to immune challenges and excess inflammation, including mitochondrial activity [49], responses to pathogenic viruses [50] and in protection of the fetus during live birth [51]**.**

Over evolutionary time, these functions were supported by the emergence of cardiovascular, digestive, renal, respiratory and reproductive systems. At the cellular and organismic level, peptide-based systems became components of the capacity to sense and respond to safety versus danger [52], as well as permitting communication and coordination among individuals [53]. The interactive functions of oxygen and OT were managed in part at the cellular level by the early evolution of ancient mitochondrial and cellular homeostatic processes, including the capacity to regulate inflammation oxidation [15,17,54]. Networks among these systems also permitted communication and coordination within systems and even between individuals [30,55,56].

## 7. Oxytocin

In its canonical form OT, as well as its paralogous sibling nonapeptides, are composed of nine amino acids, structured around a six amino acid ring and a three amino acid tail (table 2). The rings in these molecules are formed by sulfur bonds at two cysteine molecules. The ring configuration could make OT more stable and less likely to be either broken down into fragments or to become bound to other molecules. Furthermore, when OT-like peptides appear in linear forms they have exposed cysteine residues, capable of bonding to other molecules [32]. It has been suggested in this form OT-like peptides may be particularly effective in countering inflammation [33].

Two modern families of nonapeptides exist, both originating from a common ancestor (vasotocin) [14]. Based on an updated genomic analysis, a recent paper by Theofanopoulou *et al*. [19] argues for a simpler nomenclature for the family of peptides containing OT and VP, describing these peptides as the oxytocin–vasopressin (OT–VP) system.

In the vertebrate, OT–VP family of nonapeptides, vasotocin, VP and mesotocin differs from OT by either one or two amino acids (table 2). These slightly structurally different molecules can have markedly different, sometimes antagonistic functions [43] (table 3). They also may have different consequences for binding to receptors [57]. Different chemical properties of these molecules also affect their capacity to influence subcellular signalling [58] and the management of inflammation and pain [39,54].

**Table 3.** Hypothesized functional changes in oxytocin, vasopressin and CRH (and selected receptors), in the context of evolution and coping with stressors.

hypothesized functions	oxytocin (OT) and oxytocin receptors (OTR)	vasopressin (VP) and vasopressin receptors (VPRs)	CRH and CRH R1
*evolutionary history*	modern–mammalian OT 250–200 Ma	primitive vertebrates VP 300–350 Ma (vasotocin-like much earlier)	primitive early vertebrates (other CRH family peptides were much earlier
*life history strategy*	SLOW	FAST	FAST
*survival strategies*	cooperative–social	defensive–individual protective aggression	defensive–individual
*social behaviours*	approach context-dependent	approach or avoidance context-dependent	approach or avoidance context-dependent
*mobilization anxiety or fear*	anxiolytic and fear reducing Immobility without fear	anxiogenic (VPRs)	anxiogenic (CRH R1)
*acute stress*	increase—OT	increase—VP amplifies effects of CRH	increase in hypothalamic–pituitary–adrenal axis activity amplifies effects of VP
*chronic stress*	increase in OT; especially in females	increase in VP; especially in males	increase in CRH and catecholamines; especially in females
*inflammation*	anti-inflammatory primarily	pro-inflammatory primarily	anti-inflammatory initially pro-inflammatory over time
*autonomic nervous system* [30,55]	myelinated parasympathetic and sympathetic nervous systems	sympathetic nervous system and unmyelinated parasympathetic nervous system	sympathetic nervous system and unmyelinated parasympathetic nervous system
*early life experience; epigenetic*	early nurture increase OT–OTR	early neglect or abuse may increase VP–VPRs	early adversity may increase CRF–CRF R1

Canonical OT is a structurally conserved molecule. However, in New World primates, there is between species variation, owing to a single amino acid substitution at position 8 of the OT molecule; this difference has been related to the capacity for binding to OT and VP receptors and also for social behaviour in New World primates [59,60]. Amino acid substitutions in the ring or tail of OT–VP family molecules have functional effects on receptor binding. For example, the composition of the six amino acid ring formed by disulfide bonds between cysteine molecules (positions 1 and 6), has been implicated in coupling with G-protein coupled receptor (GPCR) receptors while the three amino acid tails (positions 7–9) have been implicated in ligand binding to the receptor [60].

Although precursors of the OT–VP family of peptides can be traced to over 700 Ma, this class of molecules had already begun to evolve before the last common ancestor of invertebrates and vertebrates [61]. Genes for vasotocin-like molecules existed in the ancestors of primitive fishes [62]. Mesotocin evolved around 400 Ma in non-eutherian tetrapods and birds [14]. Mesotocin is associated with high levels of sociality and social bonding found in birds [63], suggesting that this functional feature of the OT-like peptides began to emerge long before the evolution of mammals. However, the ‘modern’ OT peptide first appeared associated with the evolution of mammals, permitting lactation [4]. Functional variations in the mesotocin-OT like family of peptides also may have contributed to the evolution of species-typical traits, including patterns of social dependency [64] and contributing to longevity [23].

## 8. The oxytocin receptor

The effects of OT and other neuropeptides depend on their capacity to bind to co-evolved, ancient receptors. Receptors selectively sensitive to OT are critical to understanding its functions and many detailed reviews exist describing the OTR [65,66] and its functions [3,4]. These receptors are found throughout the body, including the immune system and are essential to specifying the functions of OT [67], as well as its interactions with other peptides [43].

The primary OTR is a GPCR which transduces information across cell membranes, with consequences for subcellular signalling. In addition, OT can indirectly influence the function of other receptors. For example, OT serves as a positive allosteric regulator of opioid receptors [68,69]. In addition, OT regulates gaseous transmitters, including hydrogen sulfide (H_2_S), nitric oxide (NO) and CO_2_, with generally protective effects on tissues throughout the body [26,27].

Cell membrane receptors for OTR have consequences for effects of OT throughout the body [27,70]. These are capable of coordinating behaviour and reproduction with environmental demands including stressful conditions. The expression of OTRs also plays a major role in the identification of tissue targets for OT, including specific cell types throughout the body [67]. Multiple examples also exist of consequences for OT, acting via the OTR, on cells that are components of the immune system [71]. For example, among the cells and tissues throughout the body expressing OTR are glia [72,73], macrophages and monocytes [74,75].

## 9. The dynamic nature of oxytocin

OT was originally believed to be a ‘female reproductive hormone’ with no known function in males [76]. This notion greatly underestimated OT's physiological importance [77]. Furthermore, it was thought that levels of OT in the body were very low [78]. However, using mass spectrometry, the concentrations of OT have been described as ‘startlingly high’ [79], at least when compared to estimates from antibody-dependent assays. It was also assumed that OT could only be accurately measured after ‘extraction’ [80]. The dynamic nature of sulfur bonds may allow OT to adhere to other thiol-rich compounds, such as glutathione, making OT both difficult to study and to measure [32,81,82]. However, various functions have been described for the linear forms and fragments of OT-like molecules; these may occur when their rings are open or when these molecules are being broken down into fragments (reviewed in [3]). Controversy still exists around the best methods for precisely measuring OT ‘levels’ [83].

It was also generally accepted in the past that OT could not pass through lipid membrane barriers, including the placenta and blood–brain barriers. However, using the carrier protein known as RAGE (receptor for advanced glycation end products), OT does cross lipid membranes. RAGE is also a component of the immune system and best known for its role in managing cytokines and inflammation [84,85].

OT can be upregulated at sites of inflammation. For example, the levels of OT measured in ovarian tumours may be 200 times greater than in blood [86]. Bodily fluids, and especially blood, may be distribution mechanisms allowing OT to be dispensed as needed. Local production of OT also may contribute to OT's effects in regions, such as the brain and uterus or at infections. Furthermore, OT can act as a signalling molecule for other functions including the timing and long-term consequences of the birth experience [17,87–89].

OT is not a rare molecule, but apparently is relatively abundant [32] with functions in both sexes. OT, or even fragments of OT, may affect functions throughout the brain and body [33,90]. Moreover, OT may be dispatched as needed to influence inflammation or infection, perhaps through fast acting gases such an H_2_S, NO and CO_2_ [27]. These findings also remind us of the evolved and dynamic partnerships between OT and basic elements including oxygen and sulfur, which are now being appreciated.

The effects of OT are most apparent during responses to challenge [4] and typically occur against a functional background of interactions between VP and CRH. A few of the general differences and interactions among the effects of the OT, arginine VP and CRH systems are summarized here and in table 3.

## 10. Oxytocin and the hypothalamic–pituitary–adrenal axis

The OT molecule has many dynamic relationships with more ancient hormones, including VP and CRH. Early versions of CRH/urocortin-like molecules and the substrates for a primordial hypothalamic–pituitary–adrenal (HPA) axis also had evolved by 541 Ma [91]. Precursors to contemporary peptide molecules are believed to have existed when the first vertebrates emerged, sometime before 443 Ma. Peptide molecules and their receptors, as well as steroids (such as mineralocorticoids and glucocorticoids) played a central role in the physiological transition from aquatic to terrestrial environments [92]. These physiological and behavioural adaptations continue to exist in modern vertebrates as components of physiological and behavioural defence and reproduction.

VP generally synergizes with CRH and in some cases other pro-inflammatory factors, to allow rapid responses to acute stressors, including mobilization, vigilance and agonistic behaviour. Both CRH and VP are called ‘stress hormones’ and are major components of the HPA axis with their own complex dynamics in the management of challenges [93].

In general, OT serves to downregulate the HPA axis and inflammation. OT is capable of inhibiting the transcription of genes regulating the expression of CRH in the hypothalamus [58]. OT and CRH are colocalized and released together facilitating the capacity to manage threat versus safety [94]. Research in animal models suggests that OT, including interactions with VP and CRH, is central to the capacity to accurately anticipate and respond to threats [95]. Over-reactivity to threats, real or imagined, could also contribute to anxiety and depression which OT may mitigate [58].

Primary among OT's partners in dealing with challenge is its paralogue, VP. OT–VP and their receptors interact as an integrated and adaptive system. In some cases, OT–VP may have similar effects. However, depending on context, the effects of OT–VP, possibly owing to interactions with each other's receptors, also can appear to be in opposite directions [43,96,97].

The sometimes antagonistic and sexually dimorphic relationship of OT–VP has been detailed elsewhere [3,4,43,98]. In general, it seems that OT–VP are synergistic during acute stress. However, during chronic situations, and especially in a context of safety and social connection, OT also may have the capacity to override the actions of VP or CRH, allowing growth and restoration. Less well understood are OT's interactions with peptides in the CRH family [94] and their receptors [99]. Urocortins are components of the CRH family of peptides and through effects on the CRH R2 receptor urocortins may have effects that resemble those of OT. The functional nature of the OT–CRH–urocortin interactions deserves deeper study [100].

## 11. Acute and chronic stress

Historically the study of acute, often transient experiences has dominated the science of stress biology. Laboratory studies of the HPA axis typically included measures of CRH, anterior pituitary hormones, adrenal steroids and their receptors. However, acute stressors act against the background of chronic stress, across a lifespan and sometimes over generations. Moreover, the neurobiology of reactions to acute versus chronic stressors is not identical and may differ in males and females [101–104]. Theories regarding stress and homeostasis also have gradually expanded to include the concept of anticipation of future needs, allostasis and allostatic load [105,106]. Especially during chronic situations, the emotional states associated with perceived safety may be critical to allow an accurate assessment of risk or threat, thus reducing or enhancing fear [30,43,94].

In mammals OT–VP are both released under conditions of acute challenges, including birth [88], intense exercise [107], sexual orgasm [76,108], severe pain or shock-trauma [27,109] and sodium challenge [110]. In all of these cases at least one of the roles for OT may be to return to homeostasis, while also predicting future allostatic demands [106], possibly in partnership with CRH [94].

Molecules in the CRH system have a complex capacity to manage both behavioural and physical challenges [100]. However, the CRH family of peptides and their receptors predate the pre-Cambrian period (table 1) [91,99]. In this context, the interactive capacity of OT–VP to affect each other could play a role in the management of both acute and chronic challenges [43]. These interactions are often sexually dimorphic, and VP appears to be of particular importance in males [102], while catecholamines are more important in chronic stress in females [103].

In general, VP and CRH support comparatively primitive defence strategies including aggression, immobilization with fear and pro-inflammatory responses to challenges, stressors and traumas. Under chronic stress OT's capacity to downregulate defence systems may be especially relevant, allowing social and psychological safety to modulate both the HPA axis and emotional reactivity. Evidence for this comes from studies of animals that are deficient in OT or the OTR, which are less able to manage stressful experiences of various kinds including events that challenge physiology [111] and behaviour [77,112]. OT may have particular importance in response to social challenges, including being protective during social isolation [113–117].

As nervous systems and immune systems became more complex, more intricate strategies of dependence and co-regulation emerged. However, even in modern mammals, OT can appear to be a silent partner, modulating and sometimes downregulating other defensive processes with effects mediated by neural, endocrine and immune systems [4]. A growing literature in humans also suggests that responses to OT are variable and may be adaptive or apparently ‘negative,’ especially in individuals expressing clinical disorders or with a history of trauma [45]. This could be a direct consequence of OT, but also leaves open a possible role for interactions with VP or CRH (and their receptors), especially in what are sometimes described as paradoxical or adverse effects of OT (reviewed in [3,43]). The capacity of OT and the OTR to suppress chronic inflammation probably has a major role in coordinating the benefits of sociality [118] and perceived safety with health [3] and longevity [23,30].

The presence of the mammalian OT–OTR system, through the capacity to modulate social behaviour as well as physiological responses to stress, oxidation and inflammation, may have a major role in permitting species differences in longevity (reviewed in [23]). We also hypothesize that interactions between cellular metabolism and the OT–OTR system played a major role in allowing the emergence of ‘slow life history’ strategies [45], which are particularly important in highly social species, such as humans [18].

## 12. Oxytocin, mitochondria and cellular respiration

The original source of mitochondria is believed to have been bacteria that invaded the unicellular ancestors of eukaryotic organisms, carrying with them a unique genome [119]. The genomes of mitochondria were reproduced and transmitted across generations, usually passed in the maternal line. Picard & Sandi [53, p. 599] have suggested that mitochondria exhibit features of social behaviours including ‘shared environments, communication, group formation, synchronization among members, interdependence and specialization and division of labour.’ Thus, even in mitochondria it is possible to detect bidirectional sociality. The relationship between mitochondria and the cells that host them creates a special form of social interaction known as endosymbiosis.

In addition to functions associated with cellular metabolism, mitochondria also must balance cell danger responses and inflammation [15,49]. Mitochondria play multiple roles in inflammatory cell activation through the production of ROS, mitochondrial energy production and the mitochondrial unfolded protein response (UPR^mt^); all of these processes seek to restore homeostasis under conditions of cellular stress. The UPR^mt^ is similar to the unfolded protein response of the endoplasmic reticulum (UPR^ER^) (see below) and is enacted when there is mitochondrial dysregulation of protein import mechanisms and folding; this occurs owing to the depletion of mitochondrial DNA, dysfunctions in oxidative phosphorylation, inhibition of protein folding machinery and/or a buildup of ROS.

Oxygen supported the existence of multi-cellular organisms**.** However, the use of oxygen as one of the primary substrates for life required cellular mechanisms for generating this energy that were different from those in unicellular organisms. For instance, the transition from anaerobic respiration at the cellular membrane to aerobic respiration at the mitochondrial membrane involved a shift from sulfur-based compounds to oxygen as a means to generate cellular energy stored as ATP.

While the extraction of energy from glucose occurs in the first stage of aerobic cellular respiration, known as glycolysis, oxygen is required for the final stage of aerobic respiration, i.e. oxidative phosphorylation. During this final stage, the co-enzymes NADH and FADH generate significant amounts of ATP; this occurs in the presence of oxygen through the transfer of electrons along the electron transport chain within the membrane of mitochondria. In a sense, mitochondria serve as rechargeable storage batteries for the cell and these functions are sensitive to OT.

OT has indirect effects on mitochondrial function via the UPR^ER^, autophagy and ROS modulation (described below). OT also may more directly mediate the effect of cellular, environmental or social challenges, especially during development, by directly affecting mitochondria. In an early example, Lehninger & Neubert [31] demonstrated a role for OT in causing mitochondrial swelling via water uptake. Water uptake and extrusion in mitochondria are essential for mitochondrial volume homeostasis and are believed to contribute to oxidative capacity, ROS production, intracellular signal transduction and mitochondrial fusion and fission [120]. Lehninger & Neubert [31] found that OT (as well as VP and insulin, which also contain cysteine bonds) induced mitochondrial swelling and that their actions were potentiated by reduced glutathione. Furthermore, they hypothesized that it was the disulfide groups on these hormones that were active components driving water uptake. The addition of ATP reversed OT while OT plus glutathione induced mitochondrial swelling.

Among the recent examples of direct effects of OT on mitochondrial function are those observed in male mice subjected to postnatal maternal separation stress [121]. As adults, these males are characterized by a depressive-like behavioural phenotype that is accompanied by impaired mitochondrial function, reduced anti-oxidant activity, increased oxidative stress and increased pro-inflammatory markers. OT mitigated the effects of early life stress on mitochondrial function by decreasing ROS formation and NO levels and increasing ATP and levels of the anti-inflammatory molecule glutathione. Remarkably, exogenous OT treatment also rescued the behavioural phenotype in this model of early life stress.

In another model, the oxygen-glucose deprivation (OGD) paradigm, rat primary neural cells display decreased mitochondrial activity. Exposure to OGD also increased extracellular levels of high-motility group Box 1 (HMGB1) [122], a chromatin protein that can elevate ROS as well as activate microglia through RAGE. Treatment of the primary neural cells with OT before OGD exposure increased mitochondrial activity, decreased HMGB1 levels and increased cell viability, effects consistent with an anti-inflammatory and neuroprotective role for OT during hypoxic-ischaemic events.

OT also has been shown to induce an acute depolarization of the mitochondrial membrane potential in isolated myometrial cells. This is probably owing to an increase in the intracellular calcium concentration by OT and the subsequent stimulation of ATP synthase, an enzyme responsible for increases in ATP energy stores [123].

More recently OT has been shown to protect mitochondrial function in peripheral immune cells from postpartum women who experienced severe childhood maltreatment [124]. Previous studies have shown that mitochondrial respiration, measured by cellular oxygen consumption, was higher in peripheral blood mononuclear cells (PBMCs) from women who had experienced greater severity of childhood maltreatment. This increased mitochondrial respiration was positively associated with increases in oxidative stress, ROS and cytokine production in peripheral blood samples, as well as telomere shortening within memory cytotoxic T cells. Notably, cortisol function was negatively associated with telomere length while OT was positively associated with telomere length [125]. In a follow-up study, higher cortisol levels were associated with increased oxygen consumption relative to basal mitochondrial respiration in PBMCS taken from individuals with significant levels of childhood maltreatment, while higher OT levels were associated with decreased oxygen consumption relative to basal mitochondrial respiration [125]. Taken together, these findings suggest that OT, by reducing the mitochondrial respiration of immune cells, may confer resilience under conditions of extreme stress [126].

## 13. Cellular stress and superoxide formation—adaptive or deleterious?

With the shift to oxygen-based electron transfer for ATP production came oxygen-based chemistry for multiple cellular processes. Superoxides are oxygen-containing free radicals such as O_2_^−^ and hydrogen peroxide (H_2_O_2_). Their generation from O_2_ molecules is essential for driving cellular homeostatic processes, such as autophagy and antimicrobial defence. Autophagy is a collection of cellular processes that are important for normal ‘housekeeping’, such as cell growth, the clearance of aggregated or misfolded proteins, the degradation and recycling of cellular organelles and molecules, and cell death [127]. Autophagy has adaptive roles in nutritional starvation. For instance, the O_2_^−^ -initiated autophagic degradation of amino acids and fatty acids during starvation serves to provide nutrients and/or generate ATP for cellular survival [128,129]. OT is a major regulator of autophagy as Klein *et al*. [130] have shown in the newborn gut; importantly OT in colostrum stimulates autophagy, protecting gut epithelial cells against the stress of amino acid insufficiency experienced between birth and first feeding.

Methods for overcoming uncontrolled defence, including anti-inflammatory and anti-oxidant processes [49], were essential to the evolution of contemporary organisms in an oxygen-rich environment. For example, breaking the bonds in oxygen also can create free radicals, energetic molecules containing unpaired and thus unstable molecules. These molecules are responsible for oxidative stress and inflammation. At high levels, reactive molecules produced during this process, such as H_2_O_2_, are damaging to cells. As described here, anti-inflammatory peptides, including OT, help to create a chemical environment that can regulate this cascade [75,131].

Superoxides also play a crucial role in the destruction of pathogenic bacteria that enter the body of an organism [132,133]. Upon immune stimulation, phagocytes (monocytes, neutrophiles, eosinophiles and macrophages) undergo a respiratory burst that involves the oxidation of O_2_ to O_2_^−^ and H_2_O_2_. The superoxides created from this burst are packaged into phagocytes and participate in bacterial killing upon phagocytosis. However, excessive superoxide production in either an acute or prolonged time frame can have damaging consequences as it can trigger autophagic cell death programmes and significantly increase inflammation and oxidative stress in cells and tissues [134]. Thus, OT may serve as a break on multiple aspects of inflammatory and oxidative stress pathways, protecting against cellular damage, especially under conditions of chronic stress.

As one example, within a model investigating cardiac homeostasis and pathophysiology, OTRs were identified on macrophages and monocytes and OT was shown to inhibit NADPH-superoxide production and the release of pro-inflammatory cytokines from these cells [74]. OT also inhibited myeloperoxidase (MPO) activity linked with neutrophil infiltration in models of significant gastrointestinal injury as well as following hepatic ischaemic-reperfusion (reviewed in [15]). MPO, a peroxidase expressed within neutrophils and monocytes, generates reactive O_2_ intermediates, such as hypochlorous acid, that have significant antimicrobial (i.e. bacteria killing) activities [135]. However, at high levels these intermediary molecules can increase oxidative stress and cell damage. These studies suggest that OT can dampen the formation of ROS within immune cells that are responding to inflammation and bacterial antigens, increasing the adaptive response to cellular stressors.

Another process that is an integral component of cellular homeostasis and the response to cellular stress and damage is the UPR^ER^. The endoplasmic reticulum (ER) is a cell organelle found in eukaryotes that functions to synthesize and properly fold proteins, modulate intracellular Ca^2 +^ concentration, and synthesize lipids and cholesterol. When cells are subjected to an increase in oxidative stress, superoxides, and/or pro-inflammatory cytokines, a buildup of unfolded or misfolded proteins can occur during protein translation, causing ER stress. The UPR^ER^ will be activated and engage signalling systems to halt protein translation and reduce the unfolded/misfolded protein load in the cell as a means of restoring cellular homeostasis (reviewed in [15]). OT has been shown to play an integral role in regulating various effector molecules of the UPR^ER^ to dampen cellular stress in intestinal cells and tissues while misfolded proteins are degraded and cleared [130,136]. In addition, OT also has been shown to inhibit the PI3 K/Akt/mTORC1 pathway involved in protein translation within cultured intestinal epithelial cells, providing a brief relief from protein synthesis, while cells clear damaged proteins [137].

It is interesting to note that OT is an important signalling molecule for the cellular processes of autophagy and the UPR^ER^ which are triggered by an increase in cellular/oxidative stress and bacterial invasion. These conditions arise naturally in the newborn gut due to the birth process. Thus, the precise release of OT during labour and with each bout of lactation is probably adaptive to reducing cellular stress in enterocytes that line the lumen of the gut, as microbes colonize the gut at birth and during the early postnatal period of breastfeeding [17,130].

## 14. Birth and oxytocin: the benefits of oxytocin under reduced oxygen

Particularly important to both survival and reproduction in mammals is the capacity of OT to moderate potentially stressful conditions, including reductions in oxygen associated with birth. For example, the process of parturition is considered an inflammatory event in which offspring are exposed to hypoxia-like conditions through a reduction in blood flow that decreases the supply of glucose and oxygen to fetal tissues [87,138]. This reduction in fetal blood supply occurs through uterine contractions and compression of the umbilical cord, placenta and fetal head during the birth process. OT, a hormone uniquely associated with stress at the time of birth [17], protects the fetal brain against hypoxia by initiating a dramatic switch in GABA signalling within neurons [139]. The depolarizing action of GABA drives neural activity in fetal and early postnatal life under ample oxygen supply [140]. However, during the birth process, OT switches GABA signalling to hyperpolarization through a reduction in intracellular Cl^−^ ([Cl^−^]_i_) [87,140]. Because maintaining high levels of [Cl^−^] in immature neurons requires significant energy in the form of ATP, the OT-mediated reduction in [Cl^−^] serves to reduce the metabolic demand of neurons for ATP during this acute period of hypoxia. Furthermore, this process has developmental consequences that may be sexually dimorphic [141], with the potential to influence vulnerability in later life to social disorders such as autism [139].

Aerobic cellular respiration is a collection of metabolic reactions performed by cells to convert oxygen or nutrients into ATP. As mentioned above, oxygen is required for the final stage of cellular respiration known as oxidative phosphorylation, which involves the transfer of electrons down the electron transport chain within the inner mitochondrial membrane. This electron transfer pumps H^+^ ions from the matrix to the intermembrane space, establishing a proton gradient that is used by ATP synthase to generate ATP molecules. The role of oxygen in this process is to accept electrons at the end of the electron transport chain, which serves to split the oxygen molecule into two and allows it to take up H^+^ to form two water molecules. Importantly, if adequate oxygen is not present for oxidative phosphorylation, the electron transport chain does not run and ATP levels plummet, increasing the vulnerability to irreversible cellular damage.

In the acute phase of OGD of neurons, termed adaptation, cells attempt to conserve energy by reducing glutamatergic transmission and undergoing cell hyperpolarization. If hypoxia continues, energy stores become depleted, and neurons enter anoxic depolarization (AD), a process characterized by the loss of ionic gradients, decreasing pH, and the activation of intracellular cascades that are responsible for increased cytokine and ROS production, elevated levels of oxidative stress and the initiation of cell death pathways owing to the progressive and uncontrollable depolarization of neurons [87,134,140].

In a model of anoxia-aglycemia that recapitulates birth conditions, blocking OT signalling through OTR antagonism accelerates the onset of AD in fetal brain tissue. This lends support to the idea that OT signalling, through the hyperpolarization of neurons, reduces metabolic demand for ATP and thereby increases neuronal resistance to birth-associated anoxia [87,140].

OT has additional protective functions during the hypoxic-like conditions at birth. Hypoxia/ischaemia events are characterized by increases in pro-inflammatory microglial signalling, oxidative stress and ROS. For example, OT has been shown to dampen oxidative stress and microglial/macrophage inflammatory cascades involving the release of pro-inflammatory cytokines (including interleukin-6 (IL-6) tumour necrosis factor alpha (TNFa) interleukin1-beta (IL-1b) following inflammatory and immune challenges [13,72,75,142]); these are probably additional adaptive functions of OT during the acute phase of parturition.

Under prolonged ischaemic conditions characterized by free radical formation, lipid peroxidation and oxidation, OT may also act as a potent anti-oxidant to delay the onset of AD in neurons. An examination of the relationship between chemical structure and anti-oxidant activity demonstrates that peptide hormones such as OT can scavenge free peroxyl radicals and inhibit low density lipoprotein oxidation and lipid peroxidation of cell membranes [143]. OT, acting as an anti-oxidant, has also been shown to inhibit NADPH-dependent superoxide activity produced by vascular cells, macrophages and monocytes in a model of cardiovascular disease [74]. OT can also act as a powerful anti-oxidant by inhibiting lipid peroxidation and MPO activity while increasing glutathione levels in models of colonic inflammation, gastric injury and hepatic ischaemia-reperfusion [144–146].

## 15. Ischaemic stroke: another model of reduced oxygen and oxidative stress

OT has also been shown to be neuroprotective following ischaemic stroke, a condition that may lead to increased oxidative stress and the initiation of pro-inflammatory cascades by microglia. Within an adult mouse model of ischaemic stroke, male mice subjected to focal cerebral ischaemia had a reduction in infarct size, an increase in anti-oxidant activity (measured by elevations in glutathione peroxidase (GPx)) and a decrease in oxidative stress (measured by an elevation in the ratio of reduced glutathione to oxidized glutathione (GSSG)); these beneficial effects were only seen if animals were socially housed rather living in isolation [142]. Importantly, social housing released endogenous OT and pretreatment with an OTR antagonist blocked these effects in the socially housed mice, while pretreatment with exogenous OT induced the protective effects in socially isolated mice. Because microglial activity is associated with increased ischaemic damage and OT was shown to reduce lipopolysaccharide-induced activation of cultured microglia from the social isolated animals, it was proposed by Karelina & DeVries [142] that OT provides neuroprotection from ischaemic stroke through the suppression of microglial reactivity and downstream pro-inflammatory cascades.

## 16. Oxytocin educates the immune system

Inflammatory processes including those involving cytokines predate the evolution of bilateria. These conserved processes formed a signalling network, including both inflammation and adaptive immunity and capable of supporting survival in response to both acute and chronic challenges [147]. Many forms of life, including plants, have mechanisms for regulating oxidation. However, as described here pro-inflammatory cytokines, initially protective against acute damage in excess are potentially dangerous, a condition which came to be called a ‘cytokine storm.’ Methods for regulating uncontrolled defense, including adaptive anti-inflammatory and anti-oxidant processes were essential to the evolution of contemporary organisms.

The role of OT in the immune system has been recently reviewed elsewhere [42]. Briefly, among the cells and tissues throughout the body expressing OTR are microglia [71,148], macrophages and monocytes [74,75]. For example, astroglia in the central amygdala express OTR with the capacity to influence emotional and behaviour effects of OT [73]. The generation and packaging of superoxides and free radicals within immune cells can also provide a means by which free radicals could be delivered to and kill pathogenic bacteria invading the host. The expression of OTRs on these cell types probably served as a break on immune cell activation and the excessive generation of free radicals in chronic inflammatory diseases states.

In the context of adaptation, the acquired immune system, including the thymus, must be programmed for ‘self-tolerance.’ As reviewed by Geenen *et al*. [149], OT plays a critical role in the ‘education’ of the thymus and may be an essential element in the development of auto-immune disorders [150]. Given the prevalence of auto-immune diseases and its relationship to early life trauma, especially in women, this is a topic that deserves additional attention.

## 17. Oxytocin regulates processes necessary for mammalian development

In larger animals, the extraction of adequate supplies of oxygen required the evolution of lungs, to capture oxygen from the environment, as well as a cardiovascular system, managing oxygen delivery to cells via rhythmic contractions of heart muscles and red blood cells [151]. Although beyond the scope of the current review, it is important to note that OT has multiple functions in the autonomic nervous system, supporting the general functions of the vertebrate body [3]. For example, as described elsewhere, both the sympathetic and parasympathetic nervous systems and their coupling are regulated by OT [3,20,152].

In mammals, autonomic process, and especially the parasympathetic nervous system, allowed the development of complex behavioural functions such as sociality and cognition and large brains [55]. OTRs are found throughout the source nuclei for the autonomic nervous system [152]. OT, through multiple levels of interaction with the autonomic nervous system, influences oxygenation, with consequences in mammals for every organ, and especially the brain. The parasympathetic nervous system, with the capacity to calm in the face of stress, may be of particular importance to sociality.

OT directly and indirectly helps to sculpt the mammalian nervous system and its functions during development and across the lifespan [20,153]. For example, flexibility in the skull is necessary to accommodate the encephalization and expansion of the neocortex. OT has several roles in bone formation, including bone mineralization and remodelling [154]; these changes are critical to the development of the mammalian skull, as well as specific features of the anatomy of human social communication, including the structure and function of the middle ear and face [55]. OT also has a role in the differentiation of stem cells [70] and can reduce cell death and promote neurogenesis, even under conditions of stress [155].

## 18. Summary: a social solution to the stress of life

OT is a biochemical component of a dynamic system that, in a context of safety, can protect the mammalian body against overreacting to chronic stressors, traumatic experiences and inflammation, including those associated with reproduction and birth. The responses to challenge that would come to be termed ‘stress’ or ‘stress responses’ were initially chemical and molecular (table 1). In the presence of higher levels of oxygen, multi-cellular organisms evolved complex neural and behavioural systems. How this actually happened can only be pieced together from fragments of information. However, we do know that in the context of living in an atmosphere dominated by oxygen the pleiotropic actions of peptides, including eventually OT, supported complex physiological systems and provided new behavioural solutions to the ‘stress of life’ (table 3). This is especially obvious in contemporary mammals, in which the actions of OT can directly and indirectly facilitate social connections, communication and an emotional sense of ‘safety’ [3,30].

More ancient peptides, including the VP and CRH families of peptides, as well as other adaptive molecules such as cytokines, also support survival under conditions of stress. However, these may favour short-term and more individualistic survival strategies (table 3). Under chronic stress or trauma, recently evolved systems can default to more primitive strategies. Especially under these severe conditions, disruptions in the homeostatic and behavioural systems supporting growth and restoration create vulnerabilities to illness and disease. Trauma or alternatively nurture, especially in early life, can epigenetic reprogramme the OT–VP system, with consequences lasting across the lifespan [45,46].

Although beyond the scope of this review, the relationship between OT and oxygen also may help to explain and eventually treat a myriad of disorders that can be influenced by excessive inflammation, stress and the absence of perceived safety; among these are almost every known disease [156], including auto-immune disorders [150] and cancer [157]. The absence of a sense of social support can have profound consequences, which are apparent in what has been called ‘deaths of despair’ [158]. However, health is not simply the absence of disease.

We suggest here that embedded in the intersecting biological consequences of peptides, including OT and the autonomic physiology of safety, are active mechanisms through which contemporary mammals use sociality to manage challenge and to adaptively allow growth and restoration [3,20,30]. We further propose that understanding the mechanisms underlying these processes is essential for creating future medicines or interventions based on emotional safety and the ‘healing power of love’ [4].

## Data Availability

This article has no additional data.
